# A Population Approach to Mitigating the Long-Term Health Effects of Combat Deployments

**DOI:** 10.5888/pcd9.110116

**Published:** 2012-02-09

**Authors:** Heather Schacht Reisinger, Stephen C. Hunt, A. Lucile Burgo, Madhulika A. Agarwal

**Affiliations:** CADRE, Iowa City VA Healthcare System; VA Puget Sound Healthcare System and University of Washington School of Medicine, Seattle, Washington; VA Connecticut Healthcare System and Yale University School of Medicine, New Haven, Connecticut; Department of Veteran Affairs, Office of Health Policy and Services, Washington, DC

## Abstract

A major focus of the mission of the US Department of Veterans Affairs (VA) is to respond to the needs of military personnel returning from war. Given the broad spectrum of the potential effects of combat deployment on the health and well being of service members, VA is increasingly oriented toward comprehensive postcombat support, health promotion, disease prevention, and proactive approaches to caring for combat veterans. This article briefly summarizes the health care needs of service members returning from Afghanistan and Iraq, describes VA's approaches to addressing their needs, and outlines VA's evolving vision for how to apply principles of population health management to ensure prompt and effective response to the postdeployment needs of veterans returning from future conflicts. At the heart of postcombat care will be population-based approaches oriented to health recovery using interdisciplinary, team-based platforms.

## Introduction

Throughout its history, a major focus of the mission of the US Department of Veterans Affairs (VA) has been to respond to the needs of military personnel returning from war. Given the broad spectrum of potential effects of combat deployment on the health and well-being of service members, VA is oriented toward comprehensive postcombat support, health promotion, disease prevention, and proactive approaches to caring for combat veterans. One goal of such care is to prevent or mitigate chronic health impairments. This article briefly summarizes the health care needs of service members of Operation Enduring Freedom in Afghanistan (OEF), Operation Iraqi Freedom (OIF) (2003-2010), and Operation New Dawn in Iraq (OND) (2010-present); describes VA's role in addressing the needs of combat veterans; and outlines VA's evolving vision for how to apply principles of population health management to ensure prompt, effective, and sustained response to the postdeployment health needs of veterans returning from future conflicts.

## Health Concerns of Veterans of Afghanistan and Iraq Deployments

Although the impact of OEF/OIF/OND is most visible in the physical injuries sustained on the battlefield, serving in combat areas affects veterans in various ways and results in a wide array of postcombat needs. Seventy-five percent of combat casualties in the current conflicts are due to explosive mechanisms of injury, primarily improvised explosive devices (1). These explosions can result in physical injury to limbs, concussion, traumatic brain injury (TBI), burns, blinding, and hearing loss. Advances in body armor and frontline medical response have increased the survival rate of soldiers with battlefield injuries (2). Sustaining such injuries, witnessing such events, and maintaining constant vigilance for such attacks also carry psychological risk. Cumulative or recurrent physical injuries and psychological traumas, along with the challenges of repetitive transitions between deployed and nondeployed status, are compounded by innumerable concomitant social and economic consequences (3).

Many injured service members have what VA has termed "polytrauma," or multiple injuries from a single event involving a complex array of discrete, often co-occurring, medical conditions with overlapping symptoms (4). Prevalence rates, which provide an inventory of injuries, often do not reveal co-occurrence of conditions, and hence belie the impact of multiple injuries on 1 person. More than 229,000 Armed Forces personnel have been diagnosed as having TBI since 2000 (5), and recent studies found that 10% to 23% of OEF/OIF service members screened positive for TBI (6,7). In the VA as of August 2011, 561,000 OEF/OIF veterans have been screened for TBI, 111,000 had an initial positive screen, and 43,000 had a confirmed TBI diagnosis after a comprehensive evaluation.With regard to mental health concerns, among Army and Marine Corps returnees from Iraq, 27% to 36% meet criteria for the broad definition of a mental health disorder 3 to 6 months after returning from deployment, including depression (10%-13%) and posttraumatic stress disorder (PTSD) (17%-25%) (8). One study found that 37% of OEF/OIF veterans receiving VA health care were diagnosed with a mental health disorder (9). Recent research has focused on the "polytrauma clinical triad," or the co-occurrence of TBI, PTSD, and chronic pain (10). In 1 study at a VA polytrauma site, a review of medical records revealed that 42.1% of the OEF/OIF patient sample were diagnosed with all 3 conditions (11). Those with all 3 conditions are also more likely to experience variability in depressed heart rates and cardiac complications (12). Although the prevalence and impact of auditory and visual impairment is understudied, 1 study of VA OEF/OIF patients found visual impairment ranged from 8.5% to 15.7%; auditory impairment, from 21.0% to 33.0%; and dual sensory impairment, from 22.7% to 35.4%; those with blast exposure had the highest rates (13). Many injured service members are recovering from numerous co-occurring conditions, including TBI, pain, amputation, visual and hearing impairment, aphasia, and PTSD, along with other mental health conditions. Besides physical injuries and mental health disorders, combat exposures increase propensity for engaging in risk behaviors, including excessive and binge drinking, verbal or physical aggression, and seeking dangerous activities (14). Finally, many returning service members face postdeployment challenges with social relationships and community reintegration (3,15). The key to providing care for this cohort of veterans has been to implement a systematic, comprehensive, and integrated approach to needs assessment and care delivery.

## VA Response: Population Health

The VA approach to health care for combat veterans is based on the standard model of population health management: the use of primary, secondary, and tertiary prevention to optimize health outcomes for OEF/OIF/OND service members (Figure). VA has developed programs and services to respond at all levels of the population health model, from veterans who have sustained devastating physical injuries to those who need and seek nothing more than readjustment advice, and for all of the veterans along that spectrum. This article focuses on the programs specific to veterans returning from Afghanistan and Iraq (Table).

**Figure.**
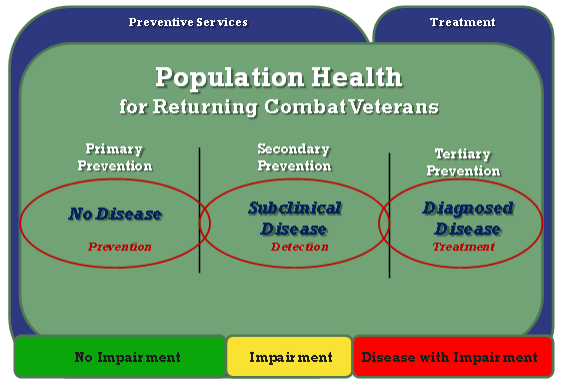
Levels of population health as applied to postcombat care. Primary prevention includes veterans with no disease who receive preventive services. Secondary prevention is screening to detect subclinical disease. Tertiary prevention is management of disease.

### Primary prevention

During the past 20 years, primary prevention has assumed a more prominent role in the VA health care system and has shifted to population-based, patient-centered primary care (16). VA uses primary prevention to promote a healthy, disease-free population of veterans, for example, through smoking cessation programs and counseling regarding diet and exercise (17). These programs have also been tailored to meet the unique needs of OEF/OIF/OND veterans. For example, VA has recently partnered with the US Department of Defense (DoD) to promote a website for tobacco cessation (www.ucanquit2.org) designed to meet the needs of younger active military and veteran populations. OEF/OIF research has found that veterans with PTSD and other mental health conditions have higher rates of tobacco use, hypertension, hyperlipidemia, and obesity compared with veterans without mental health diagnoses, so an emphasis on primary prevention is particularly relevant for this population (18).

The current transition in VA to a medical-home model of care delivery, called patient-aligned care teams (PACT), strengthens the population-based approach while focusing attention on individual patients. The objective of a medical-home model is to place the patient at the center of a health care team that examines the patient's current health status, monitors his or her long-term health and disease trajectory, and coordinates and manages his or her overall medical care. The strength of the medical-home model in supporting population-based health has been demonstrated in other health care delivery systems (19). VA's use of electronic health record (EHR) systems also contributes to its ability to support population-based health for combat veterans through assessment of outcomes stratified by a provider's or primary care team's patient panels or by patient diagnoses. Point-of-care clinical reminder systems permit standardized screening and risk assessment, and capabilities for real-time clinical decision support are evolving.

### Secondary prevention

The initial step of comprehensive care for all returning combat veterans is standardized assessments and screenings, which may reveal unacknowledged, unrecognized, and undiagnosed disease or illness. Here, the VA health care system moves to secondary prevention, or early detection and case finding of subclinical disease. All OEF/OIF/OND veterans in the VA health care system are flagged in the EHR for specific screening assessments. These screenings may help providers detect mental health conditions, such as depression, PTSD, or suicide risk. They also allow for documentation of blast exposures and prompt referrals for veterans to receive more intensive evaluation based on this history. An OEF/OIF registry has been developed to facilitate treatment, monitoring, and research related to various war-related illnesses. VA has also initiated a TBI registry to establish a database of all veterans who have symptoms possibly resulting from TBI or who have a TBI diagnosis. Secondary prevention, early detection, and case finding are strengthened by population screening, an extensive system of clinical reminders in VA's EHR, and deployment-related registries.

### Tertiary prevention

Finally, the notion of "veteran health care" generally brings to mind what is considered to be tertiary prevention in terms of the population health model: the diagnosis and management of clinical disease (both clinical management and patient self-management) to reduce long-term impairments. VA has numerous programs to treat and rehabilitate veterans with war-related injuries. The first step in treating injured service members is to ensure a smooth transition from DoD to VA health care facilities. After leaving a DoD medical treatment facility, a veteran may transition to different levels of care within VA depending on the extent of his or her injuries. Veterans who have sustained a spinal cord injury will receive care at 1 of 21 VA Spinal Cord Injury Centers until they are ready to return home (20). Those who have sustained multiple, extensive injuries may first receive care at 1 of 5 national Polytrauma Rehabilitation Centers (21). When clinically appropriate, the veteran will be transitioned to a VA medical center closer to home for continued rehabilitation and recovery. The polytrauma centers focus on interdisciplinary, integrated care; VA's national health care system and EHR allow for seamless transitions within or between VA facilities. Rehabilitation programs in local VA medical centers offer physical, occupational, and vocational therapy, as well as surgical, prosthetic, and other specialty support.

Postdeployment services are integrated for the veteran within and between programs. VA is currently undergoing a transformation in its basic platform of care to the PACT model of veteran-centered, team-based care coordinated around the needs and preferences of the individual veteran. For returning combat veterans, this transformation requires PACT alignment with mental health, social work, OEF/OIF/OND care management, suicide prevention, polytrauma, pain management, and women's health programs. Further integration of the services and resources of these programs is accomplished through the interdisciplinary, primary care–based Post-Deployment Integrated Care Initiative, which provides education, training, and support for postcombat care in VA medical centers nationwide. This initiative works closely with the Primary Care-Mental Health program, which has greatly enhanced integration of mental health care into primary care in VA. System-wide care for combat veterans is a component of the recently implemented PACT model. The core principles of all these programs include continuously improving care that is veteran-centered, team-based, case managed, and evidence-based. The objective is to assess the veteran comprehensively for postdeployment care needs (ie, physical, psychological, and psychosocial), to appropriately triage the veteran, to create an interdisciplinary care plan, and to monitor the veteran based on intensity of needs.

Encouraging all OEF/OIF/OND veterans to access VA health care and enhancing collaborative efforts between VA and other agencies are part of VA's population-based approach. The Vet Centers, originally established after the Vietnam War to provide counseling for veterans in less formal settings outside VA facilities, have incorporated OEF/OIF/OND veterans into their mission and continue to provide an open, destigmatized, and confidential place where veterans can seek help (22). Three War Related Illness and Injury Study Centers operate across the country and offer education, training, clinical services, and research support to VA facilities regarding the health effects of war (23). Finally, VA has reached out to veterans' families and caregivers, helping them get veterans access to the care they need and, more recently, providing educational, counseling, and financial support to caregivers who provide care and help veterans recover function.

## Improving Postcombat Care in the VA

Many efforts are underway for improving VA care for returning combat veterans. All combat veterans can receive no-fee VA care for potentially service-related concerns for 5 years following discharge from active duty. The transition of care from DoD to VA for veterans of the current conflicts is complicated by the fact that half of the members of the military deployed to Iraq and Afghanistan have been members of the Reserve and the National Guard. Many have been deployed more than once and in the process have experienced numerous transitions in care between DoD and VA as they shift in and out of active duty status. To help with these transitions in care, VA and DoD have collaborated on many projects, including creation of a shared medical record system. VA and DoD have developed liaison programs to help service members and their families transition from military treatment facilities to VA facilities, as well as outreach programs for service members who are not seriously injured but who may need help with reintegration. Coordination of care in these transitions between VA and DoD is necessary to avoid gaps in care. When a service member is discharged and becomes a veteran or a National Guard soldier returns home from active duty, VA seeks to begin providing comprehensive care.

VA is a national system of health care with 153 medical centers and more than 900 community-based outreach clinics, but the full range of comprehensive services is geographically dispersed and not available in every veteran's home community. To meet the distance barrier, VA is using new telehealth technologies to bring specialty care closer to the veteran.

## VA's Vision for Preparedness

VA must provide high-quality, interdisciplinary, integrated care to combat veterans to ensure they recover their optimal health and reintegrate successfully into civilian society and postwar life. VA has developed effective strategies and approaches to serve needs of veterans returning home from war. During times of peace, as an essential part of our national infrastructure of preparedness, these capabilities for postdeployment care must be maintained and ready for activation. Preparing for future conflicts will entail understanding the potential environmental agent or toxic exposures service members may face in fields of combat and remaining abreast of advances in battlefield and military technology. Our postcombat care system must include collaborative relationships among VA, DoD, and other government and community agencies, and integrated records systems to identify and track emerging symptoms, illnesses, and injuries. The system must have training plans to educate VA and community providers on needs of returnees and appropriate screening tools to detect past physical and mental health issues in new contexts. VA's doors should remain open to service members and their families who are struggling with readjustment, and VA should have the capability of quickly putting together a national response that allows recovering veterans to transition seamlessly to civilian life and to do so closer to home. At the center of preparedness for war will be a population-based approach to health recovery that uses interdisciplinary teams for postcombat care.

## Figures and Tables

**Table.** VA Programs Related to OEF/OIF/OND Postdeployment Care Categorized by Levels of the Population Health Model (Primary, Secondary, and Tertiary Prevention)

**Programs Directed at 3 Levels of Prevention**		

**Primary Prevention**	**Secondary Prevention**	**Tertiary Prevention**
Outreach to OEF/OIF/OND veterans and their families Education and training programs for communities, families, and veterans Primary care Mental health care Social work OEF/OIF/OND Care Management Program Pain Management Women Veterans Health Care Polytrauma Rehabilitation Centers Spinal Cord Injury centers Office of Public Health Health care team monitoring current health status to prevent future illness and chronic disease (eg, smoking cessation counseling, weight loss counseling) Immunizations	Screening and assessment Flagged in electronic health record (EHR) for OEF/OIF/OND veterans EHR clinical reminder with screening for posttraumatic stress disorder, suicide risk, and other mental health conditions; traumatic brain injury and blast exposure history OEF/OIF veteran registries	Liaison program for transition from military treatment facility to VA medical center Polytrauma Rehabilitation Centers Spinal Cord Injury CentersPhysical therapy, occupational therapy, and vocational rehabilitation (every VA medical center) Patient Aligned Care Teams —integrated care at all VA medical centers and community based clinics Primary care Mental health care Social work OEF/OIF/OND Care Management Program Pain Management Program Women Veterans Health Care Other specialty services
**Programs, Services, and Tools for Integrating Veteran Health Care Across 3 Levels of Prevention**		
Patient Aligned Care Teams — modeled after Patient Centered Medical Homes Electronic Health Records Post-Deployment Integrated Care Initiative — education, training, and support for postcombat care across VA War Related Illness and Injury Study Centers Vet Centers		

Abbreviations: VA, US Department of Veterans Affairs; OEF, Operation Enduring Freedom; OIF, Operation Iraqi Freedom; OND, Operation New Dawn.
